# LncRNA FAM83H‐AS1 promotes oesophageal squamous cell carcinoma progression via miR‐10a‐5p/Girdin axis

**DOI:** 10.1111/jcmm.15530

**Published:** 2020-06-24

**Authors:** Bo Feng, Gaoyan Wang, Xiaoliang Liang, Zheng Wu, Xinchen Wang, Zhiming Dong, Yanli Guo, Supeng Shen, Jia Liang, Wei Guo

**Affiliations:** ^1^ Laboratory of Pathology, Hebei Cancer Institute the Fourth Hospital of Hebei Medical University Shijiazhuang China

**Keywords:** ceRNA, FAM83H‐AS1, long non‐coding RNA, oesophageal squamous cell carcinoma

## Abstract

Long non‐coding RNAs (lncRNAs) have been well demonstrated to emerge as crucial regulators in cancer progression, and they can function as regulatory network based on their interactions. Although the biological functions of FAM83H‐AS1 have been confirmed in various tumour progressions, the underlying molecular mechanisms of FAM83H‐AS1 in oesophageal squamous cell carcinoma (ESCC) remained poorly understood. To address this, we treated human oesophageal cancer cell line Eca109 cells with TGF‐β and found FAM83H‐AS1 was notably overexpressed. In the present study, FAM83H‐AS1 was observed to be significantly up‐regulated in ESCC tissues and was associated with TNM stage, pathological differentiation and lymph node metastasis. FAM83H‐AS1 reinforced oesophageal cancer cell proliferation, migration and invasion, and participated in epithelial‐to‐mesenchymal transition (EMT) process at mRNA and protein levels. In addition, a concordant regulation between FAM83H‐AS1 and its sense strand FAM83H was detected at the transcriptional and translational levels. Furthermore, FAM83H‐AS1 could act as competing endogenous RNA to affect the expression of Girdin by sponging miR‐10a‐5p verified by RIP and luciferase reporter assays. Consequently, the study provided a unique perspective of FAM83H‐AS1 in ESCC progression, which may be considered as potential biomarker and therapeutic target for ESCC therapy.

## 1 INTRODUCTION

Globally, oesophageal cancer is the seventh most common malignant cancer type and represents the sixth‐highest incidence in cancer mortality with a poor 5‐year survival rate.^1^ Oesophageal squamous cell carcinoma (ESCC) is the predominant histological subtype of oesophageal cancer^2^ and exhibits a striking regional variation in morbidity and mortality in northern China, such as in Hebei, Shanxi and Henan provinces.^3^ Although the diagnostic and therapeutic techniques of oesophageal cancer have been largely improved in past decades, the 5‐year survival rate of oesophageal cancer remains less than 20%.^4^ Moving forward, it is essential for a more thorough understanding of the biological underpinnings of the disease and an urgent need for early detection and effective treatment regimens.

LncRNAs performed diverse functions in gene expression networks depending on their subcellular localization, such as regulating chromatin modification and gene transcription in the nucleus, and modulating mRNA stability, translation and post‐translational modification in the cytoplasm.^5^ LncRNAs located in the cytoplasm can serve as competitive endogenous RNAs (ceRNAs), which could sponge miRNAs through competition for shared miRNAs, thereby imposing an additional regulation on miRNA targets at post‐transcriptional level.^6^

Epithelial‐to‐mesenchymal transition (EMT) is a typically fundamental transdifferentiation process in development which enables cancer cell invasion, contributes to cancer stroma formation, generates stem‐like tumour‐initiating cells and increases drug resistance. Among a myriad of EMT‐regulating factors discovered in the cancer microenvironment, transforming growth factor‐β (TGF‐β) has been shown to be a potent signal to initiate and drive EMT.^7^ To identify and characterize novel factors potentially related to TGF‐β‐induced tumour aggression in oesophageal cancer, we treated human oesophageal cancer cell line Eca109 with TGF‐β and then used microarray analysis to compare RNA expression levels between TGF‐β‐treated and untreated cells, observed TGF‐β‐dependent up‐regulation of FAM83H‐AS1 (FAM83H antisense RNA1, also known as onco‐lncRNA‐3). In addition, Jiang et al^8^ identified many super‐enhancer (SE)‐associated and squamous cell carcinoma (SCC)–specific oncogenic transcripts profiled by RNA‐Seq, including FAM83H‐AS1. FAM83H‐AS1 and its cognate sense strand FAM83H are head‐to‐head located on 8q24. Natural antisense transcripts (NATs) are defined as RNA sequences that originate from complements of their endogenous sense counterparts in cis or trans.^9^, ^10^ Notably, some natural antisense lncRNAs were reported to exert regulatory effects on expression of their sense protein‐coding genes.^11^, ^12^ But the expression level and correlation between FAM83H‐AS1 and FAM83H in ESCC were not well characterized. Accumulating evidence was illustrating that FAM83H‐AS1 was overexpressed in various cancer types that promoted cell growth and metastasis by multiple molecular mechanisms,^13^, ^14^, ^15^, ^16^ while the exact mechanism of FAM83H‐AS1 in ESCC was largely unclear and its prospect as therapeutic target for ESCC was still unexplored.

In the present study, we aimed at providing an integrated analysis on the expression and correlation between FAM83H‐AS1 and FAM83H, the potential biological function of these antisense‐sense strands and downstream regulatory mechanism of FAM83H‐AS1 in the pathogenesis of ESCC, as well as its role in TGF‐β‐induced EMT.

## 2 MATERIALS AND METHODS

### 2.1 Patients and specimens

All the 67 pairs of ESCC tissues and corresponding normal tissues were taken from the surgical specimens of ESCC patients from the years of 2015 to 2017 in the Fourth Affiliated Hospital of Hebei Medical University. Informed consent was received from all patients who were not given any radiotherapy or chemotherapy before operation. According to the standard of American Joint Committee on Cancer system, histological grade was staged. Information on clinical data and clinicopathological characteristics was available from hospital recordings and is summarized in Table S1. Smokers were defined as former or current individuals smoking at least five cigarettes per day for 2 years or longer.^17^ Individuals with at least one first‐degree relative or at least two second‐degree relatives having oesophageal/cardia/gastric cancer were defined as having family history. Ethical consent was granted from the Ethics Committee of the Fourth Affiliated Hospital of Hebei Medical University.

### 2.2 Cell culture and treatment

Human oesophageal cancer cell lines Kyse150, Kyse170, TE1 and Eca109 were purchased from American Type Culture Collection and were cultured in RPMI 1640 (Invitrogen) medium containing 10% foetal bovine serum (Invitrogen) at 37°C in an atmosphere containing 5% CO_2_. The cells were treated with 10 ng/mL of recombinant TGF‐β1 (R&D Systems) for 7 days with the medium replenishment every 2 days.

### 2.3 RNA isolation and quantitative real‐time polymerase chain reaction (qRT‐PCR) assay

Total RNA from the tissues and cells was isolated using TRIzol reagent (Invitrogen) in accordance with the manufacturer's instructions. Transcriptor First Strand cDNA Synthesis Kit (Roche) was used to generate cDNA according to the manufacturer's protocol. qRT‐PCR was performed in the StepOne Real‐Time PCR System (Applied Biosystems) using GoTaq^®^ qPCR Master Mix (Promega). GAPDH and U6 snRNA were employed as endogenous controls for mRNA/lncRNA and miRNA, respectively. The relative expression level of RNAs was calculated using the 2^−ΔΔCT^ method.^18^ Each specimen was tested in triplicate. Primer sequences are displayed in Table S2.

### 2.4 Subcellular fractionation

The nuclear and cytoplasmic fractions of oesophageal cancer cell lines were isolated by PARIS™ Kit Protein and RNA Isolation System (Invitrogen) according to the manufacturer's protocol.

### 2.5 Cell transfection

The shRNAs targeting FAM83H‐AS1 and the pcDNA3.1‐FAM83H‐AS1 were designed and synthesized by GenePharma and Sangon Biotech, respectively. The miR‐10a‐5p mimics, inhibitor and negative control were purchased from GenePharma. The FAM83H siRNAs and si‐NC were synthesized by General Biosystems. Transfections were performed using Lipofectamine 2000 Reagent (Invitrogen) according to the manufacturer's protocol. The sequences of four shRNAs, three siRNAs and miR‐10a‐5p mimics and inhibitor are listed in Table S3.

### 2.6 Cell proliferation assay

The ability for cellular proliferation was detected by MTS assay and clone formation assay. The MTS assay was measured using CellTiter96^®^AQ_ueous_ One Solution Cell Proliferation Assay kit (Promega). For MTS assay, the transfected cells were seeded into 96‐well plate with 1 × 10^3^ per well. After incubation at 0, 24, 48, 72 and 96 hours, cells of each well were added with 20 μL (500 μg/mL) of MTS reagent and incubated at CO_2_ incubator for 2 hours. The optical density was measured with a microplate reader at a wavelength of 490 nm. For clone formation assay, 3 × 10^3^ cells per well following transfection for 24 hours were inoculated into a six‐well plate and regularly cultured for 1 week. More than 50 cells were considered to be one clone, and the numbers of clone were counted under a microscope.

### 2.7 Transwell migration and invasion assays

Cell migration assay was conducted using non–Matrigel‐coated chambers (Corning) with 8‐μm pore membranes. A total of 1 × 10^5^ cells per well were seeded into the upper compartment of chamber. After 24 hours of incubation at 37°C, the invasive cells located on the lower surface of the membrane were counted in five randomly selected visual fields using a Leica DMI4000B microscope. For invasion assay, the upper surface of the membrane was pre‐coated with 50 μL 1× Matrigel^®^ Basement Membrane Matrix (Corning) to form a matrix barrier; the remaining steps were used for invasion assay as described above.

### 2.8 Western blot analysis

Total proteins were extracted from transfected cells using RIPA lysis buffer containing PMSF (Solarbio) and protease inhibitor cocktail (Promega) according to the instructions of the manufacturer. The protein concentration was determined by BCA Protein Assay Kit (Multi Sciences). After mixed with loading buffer and heated at 99°C for 5 minutes, the protein lysates were separated by 10% sodium dodecyl sulphate‐polyacrylamide gel electrophoresis and transferred onto PVDF membranes (Millipore). The transferred membranes were blocked with 5% skim milk for 1 hour at room temperature and incubated overnight with the specific primary antibodies at 4°C. Subsequently, the membranes were incubated at room temperature with horseradish peroxidase–conjugated goat anti‐rabbit IgG (KPL) for 1 hour. The bands were visualized with enhanced chemiluminescence (ECL) detection reagent (Multi Sciences) by Chemi XT 4 (Syngene). The primary antibodies were used: anti‐β‐actin (AC026, ABclonal), anti‐E‐cadherin (E‐AB‐35932, Elabscience), anti‐N‐cadherin (E‐AB‐64011, Elabscience) and anti‐FAM83H (ab121816, Abcam).

### 2.9 Vectors construction

The restriction sites of pcDNA3.1‐FAM83H‐AS1 were Hind Ⅲ/EcoR I. By double digested with EcoR I and Xho I (Takara) of pSL‐MS2‐12X (Addgene), we subcloned MS2‐12X fragment into pcDNA3.1, pcDNA3.1‐FAM83H‐AS1, pcDNA3.1‐FAM83H‐AS1‐MUT, and named pcDNA3.1‐MS2, pcDNA3.1‐FAM83H‐AS1‐MS2 and pcDNA3.1‐FAM83H‐AS1‐MS2‐MUT, respectively. The sequences of FAM83H‐AS1 or Girdin 3′ UTR containing the miR‐10a‐5p recognition sites were PCR‐amplified and subcloned into pmirGLO vector (Promega) with the restriction sites of Nhe I/Xho I or Xho I/Xba I, named pmirGLO‐FAM83H‐AS1‐1 (WT), pmirGLO‐FAM83H‐AS1‐2 (WT) and pmirGLO‐Girdin 3′ UTR (WT). The point mutations of FAM83H‐AS1 or Girdin 3′ UTR binding to miR‐10a‐5p were promoted using a Q5^®^ Site‐Directed Mutagenesis Kit (New England Biolabs), named pmirGLO‐FAM83H‐AS1‐1 (MUT), pmirGLO‐FAM83H‐AS1‐2 (MUT) and pmirGLO‐Girdin 3′ UTR (MUT). Mutation primers were designed in NEBase changer (http://nebasechanger.neb.com/) and synthesized by Generay Biotech. All constructed plasmids were sequenced correctly. The primers used for vectors construction are listed in Table S4.

### 2.10 RNA immunoprecipitation (RIP) assay

Eca109 cells were cotransfected with pcDNA3.1‐MS2, pcDNA3.1‐MS2‐FAM83H‐AS1, pcDNA3.1‐MS2‐FAM83H‐AS1‐MUT and pMS2‐GFP (Addgene). After 48 hours, cells were used to perform RNA immunoprecipitation (RIP) experiments using GFP antibody (Roche) and Magna RIP™ RNA‐Binding Protein Immunoprecipitation Kit (Millipore) according to the manufacturer's instructions. IgG and SNRNP70 were respectively used as negative control and positive control.

### 2.11 Luciferase reporter assay

PmirGLO‐FAM83H‐AS1 (WT), pmirGLO‐FAM83H‐AS1 (MUT), pmirGLO‐Girdin 3′ UTR (WT) and pmirGLO‐Girdin 3′ UTR (MUT) were cotransfected with miR‐10a‐5p mimics or miR‐negative control into Eca109 cells using Lipofectamine 2000. Luciferase activity was measured with Dual‐Luciferase Reporter Assay System (Promega) at 48 hours after transfection and normalized to Renilla luciferase activity.

### 2.12 Statistical analysis

All data were expressed as mean ± SD. The significance of differences between two groups or among multiple groups was determined by Student's *t* test or one‐way ANOVA, respectively. Bivariate correlations between study variables in tissues were calculated by Spearman correlation analysis. All statistical tests were two‐sided, and *P* < .05 was considered to be statistically significant.

## 3 RESULTS

### 3.1 FAM83H‐AS1 emerges as a potential oncogenic lncRNA and is associated with clinicopathological characteristics in ESCC

**FIGURE 1:**
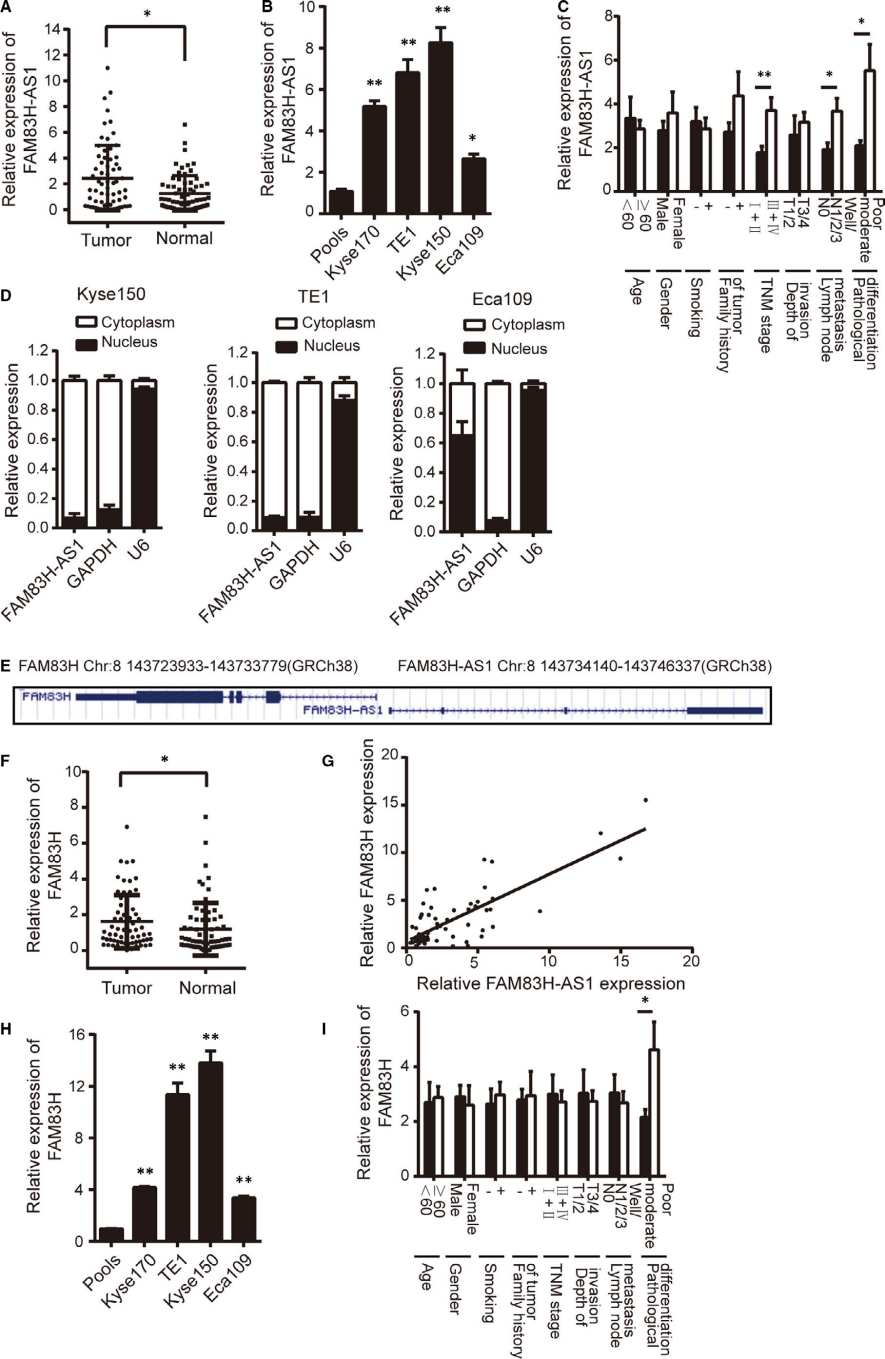
FAM83H‐AS1 and FAM83H are significantly up‐regulated and are associated with clinicopathological characteristics. A, Relative expression of FAM83H‐AS1 in 67 pairs of ESCC tissues and corresponding normal tissues confirmed by qRT‐PCR method. B, Relative expression of FAM83H‐AS1 in four human oesophageal cancer cell lines detected by qRT‐PCR method. Pools: average expression in 10 normal tissues was used as normal control. * Compared with the pools. C, Relative expression of FAM83H‐AS1 in different subgroups. D, The subcellular localization of FAM83H‐AS1 in oesophageal cancer cells. E, Schematic representation of the genomic organization of FAM83H‐AS1 and FAM83H cited from NCBI. F, Relative expression of FAM83H in 67 pairs of ESCC tissues and corresponding normal tissues detected by qRT‐PCR method. G, The correlation between FAM83H‐AS1 and FAM83H expression determined by qRT‐PCR method. H, Relative expression of FAM83H in four human oesophageal cancer cell lines detected by qRT‐PCR method. Pools: average expression in 10 normal tissues was used as normal control. * Compared with the pools. I, Relative expression of FAM83H in different subgroups. Data are shown as mean ± SD; *P < .05 and **P < .01

Based on scanning the NCBI and GEPIA data set, the relative expression levels of FAM83H‐AS1 in different normal tissues and in most of the tumour types were detected (Figure S1A,B). By evaluating FAM83H‐AS1 expression in 67 pairs of ESCC tissues and corresponding normal tissues, it was confirmed that FAM83H‐AS1 expression level was significantly elevated in ESCC tissues (Figure 1A). Additionally, the expression level of FAM83H‐AS1 in a panel of human oesophageal cancer cell lines was performed, which was remarkably higher in all oesophageal cancer cell lines, especially in Kyse150 and TE1 cells (Figure 1B). It was identified that high expression level of FAM83H‐AS1 was closely associated with lymph node metastasis, TNM stage and pathological differentiation (Figure 1C). LncRNAs have been shown to play functional roles in both the nucleus and cytoplasmic compartments. FAM83H‐AS1 was found to be predominantly located in the cytoplasm of oesophageal cancer cells by subcellular fractionation assay (Figure 1D). Coding Potential Calculator and Coding Potential Assessment Tool were further used to analyse the coding potential of FAM83H‐AS1, and no protein‐coding potential of FAM83H‐AS1 was found (Figure S1C,D).

### 3.2 FAM83H is significantly up‐regulated in ESCC patients

A NCBI search identified that FAM83H‐AS1 was in a head‐to‐head orientation relative to FAM83H (Figure 1E). As indicated by NCBI and GEPIA data set, the relative expression levels of FAM83H in normal tissues and in various tumour types were similar to FAM83H‐AS1 (Figure S1E,F). Subsequently, qRT‐PCR analysis detected increased mRNA expression level of FAM83H in ESCC tissues and FAM83H exhibited concordant co‐regulation with FAM83H‐AS1 (Figure 1F,G). Meanwhile, the expression level of FAM83H was significantly higher in Kyse150 and TE1 cells than other tested cell lines, Kyse150 and TE1 cells were selected for subsequent experiments (Figure 1H). In addition, analysis of the correlation between FAM83H expression and clinicopathological characteristics showed that FAM83H expression level was intimately associated with pathological differentiation (Figure 1I).

### 3.3 The effect of FAM83H‐AS1 and FAM83H on oesophageal cancer cell proliferation, migration and invasion

**FIGURE 2:**
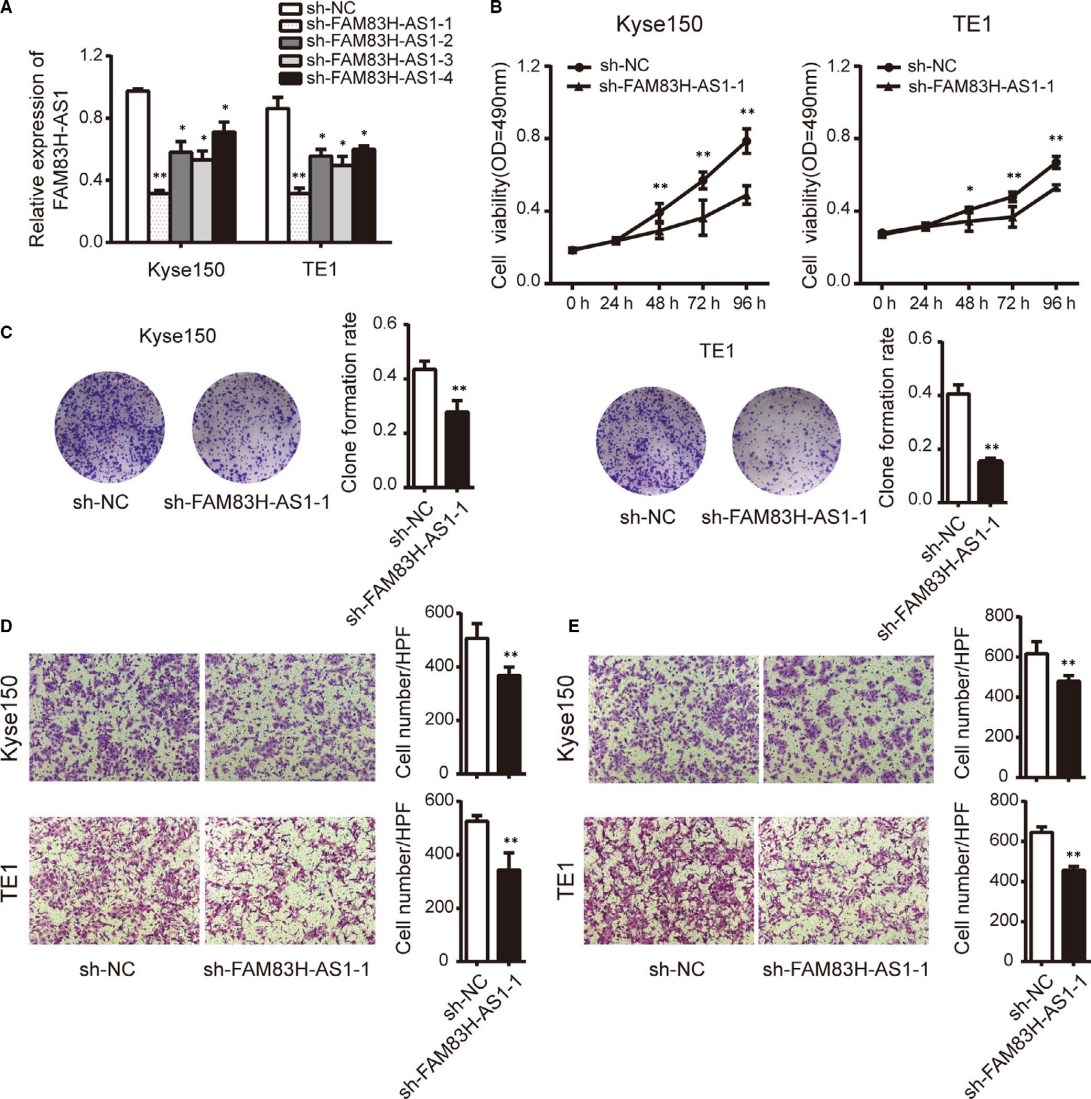
Knockdown of FAM83H‐AS1 suppresses cell proliferation, migration and invasion in oesophageal cancer cells. A, The knockdown efficiency against FAM83H‐AS1 detected by qRT‐PCR method. B, Cell proliferation was assessed using MTS assay following FAM83H‐AS1 knockdown in Kyse150 and TE1 cells. C, Clone formation assay was conducted following FAM83H‐AS1 knockdown in Kyse150 and TE1 cells. D, Transwell migration and E, invasion assays were performed in Kyse150 and TE1 cells with FAM83H‐AS1 knockdown, respectively (magnification, ×200). Data are shown as mean ± SD; *P < .05 and **P < .01

To determine the biological function of FAM83H‐AS1 and FAM83H on oesophageal cancer cells, the gain‐ and loss‐of‐function assays were performed in oesophageal cancer cells. First of all, we transfected sh‐FAM83H‐AS1 specific for FAM83H‐AS1 in Kyse150 and TE1 cells, respectively, and qRT‐PCR analysis demonstrated that the shRNAs could significantly decrease the expression level of FAM83H‐AS1 with successful transfection efficiency (Figure 2A). The sh‐FAM83H‐AS1‐1, which exhibited the most evident knockdown efficacy, was selected for follow‐up experiments. By performing MTS assay, we observed that FAM83H‐AS1 knockdown significantly inhibited cell proliferation capacity of Kyse150 and TE1 cells compared with parallel cells transfected with sh‐NC (Figure 2B). Similar growth inhibiting effect was also validated by clone formation assay (Figure 2C). By performing transwell migration and invasion assays, we then found that the migration and invasion ability of Kyse150 and TE1 cells were markedly attenuated after FAM83H‐AS1 shRNA transfection (Figure 2D,E).

As shRNA knockdown studies may be affected by off‐target effects, the impacts of FAM83H‐AS1 overexpression on oesophageal cancer cells were subsequently performed. The pcDNA3.1‐FAM83H‐AS1 was transfected into Eca109 cells which could dramatically promote the expression level of FAM83H‐AS1 in comparison with the empty vector control (Figure S2A). A series of functional experiments were performed, which validated the oncogenic role of FAM83H‐AS1 in reinforcing the proliferation, migration and invasion capacities of Eca109 cells (Figure S2B‐E).

**FIGURE 3:**
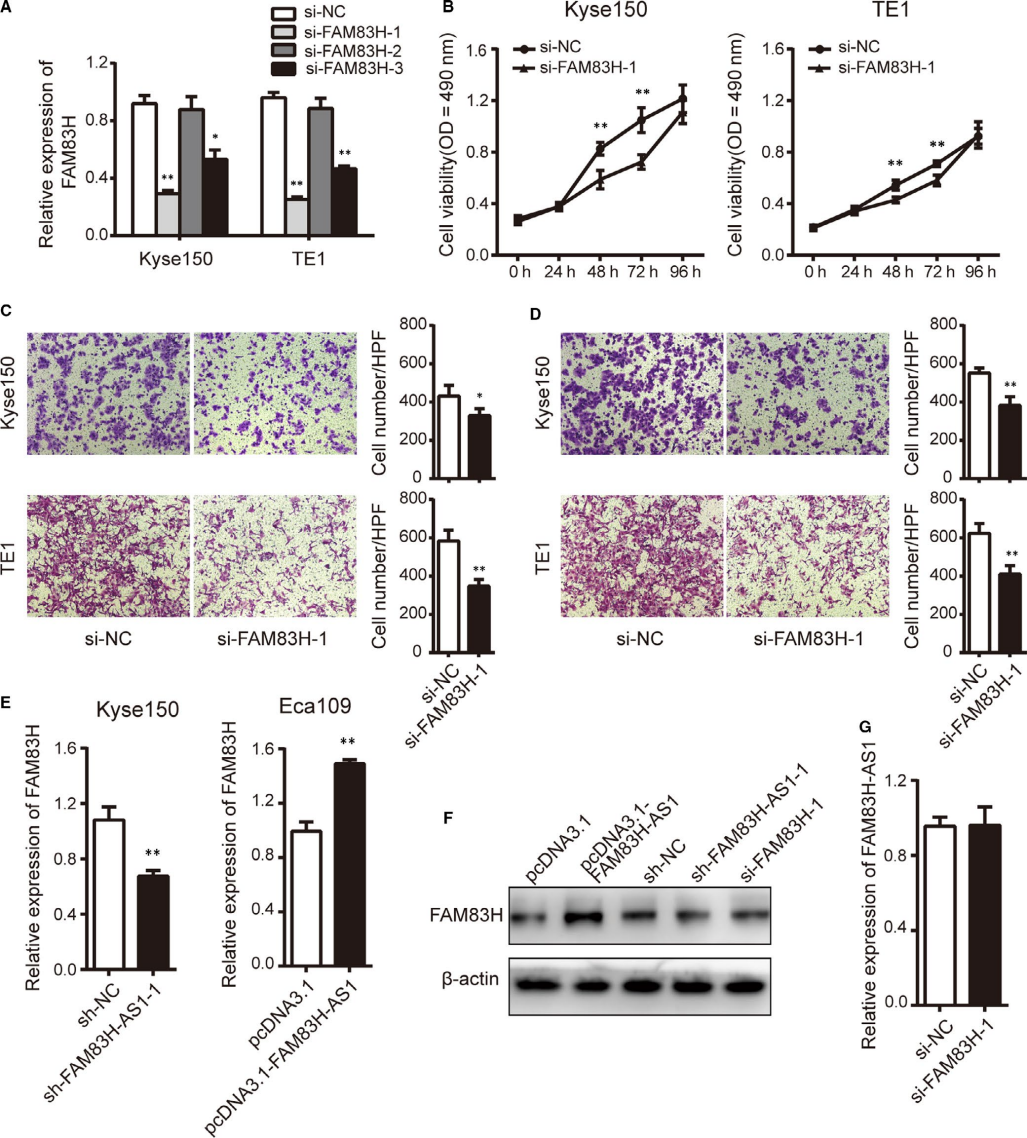
FAM83H implicates in ESCC progression and is regulated by FAM83H‐AS1 at mRNA and protein level. A, The interfering efficiency against FAM83H analysed by qRT‐PCR method. B, Cell proliferation was assessed using MTS assay with silenced FAM83H in Kyse150 and TE1 cells. C, Transwell migration and D, invasion assays were carried out with silenced FAM83H in Kyse150 and TE1 cells (magnification, ×200). E, and F, The influence of FAM83H‐AS1 on FAM83H mRNA and protein level detected by qRT‐PCR and Western blot assays. G, The influence of FAM83H on FAM83H‐AS1 expression analysed by qRT‐PCR method. Data are shown as mean ± SD; *P < .05 and **P < .01

If FAM83H in regulating biological processes was in accordance with the oncogenic role of FAM83H‐AS1, the functional experiments of FAM83H were conducted in Kyse150 and TE1 cells. We screened si‐FAM83H‐1 with high interference efficiency compared with the non‐targeting control si‐NC group (Figure 3A). As displayed in Figure 3B‐D, siRNA‐mediated FAM83H knockdown notably inhibited cell proliferation, migration and invasion, which largely phenocopied sh‐FAM83H‐AS1 inhibition in oesophageal cancer cells.

### 3.4 FAM83H‐AS1 regulates FAM83H at mRNA and protein levels

To identify the correlation between the levels of FAM83H‐AS1 and FAM83H, we assessed expression changes in FAM83H level following the knockdown or overexpression of FAM83H‐AS1. As expected, down‐regulation of FAM83H‐AS1 in Kyse150 cells decreased mRNA and protein expression levels of FAM83H. Conversely, overexpression of FAM83H‐AS1 in Eca109 cells resulted in significant increased expression of FAM83H at both mRNA and protein levels (Figure 3E‐F). However, we failed to detect any significant expression changes of FAM83H‐AS1 upon FAM83H knockdown (Figure 3G).

### 3.5 FAM83H‐AS1 is up‐regulated in TGF‐β‐induced Eca109 cells and contributes to the process of EMT

**FIGURE 4:**
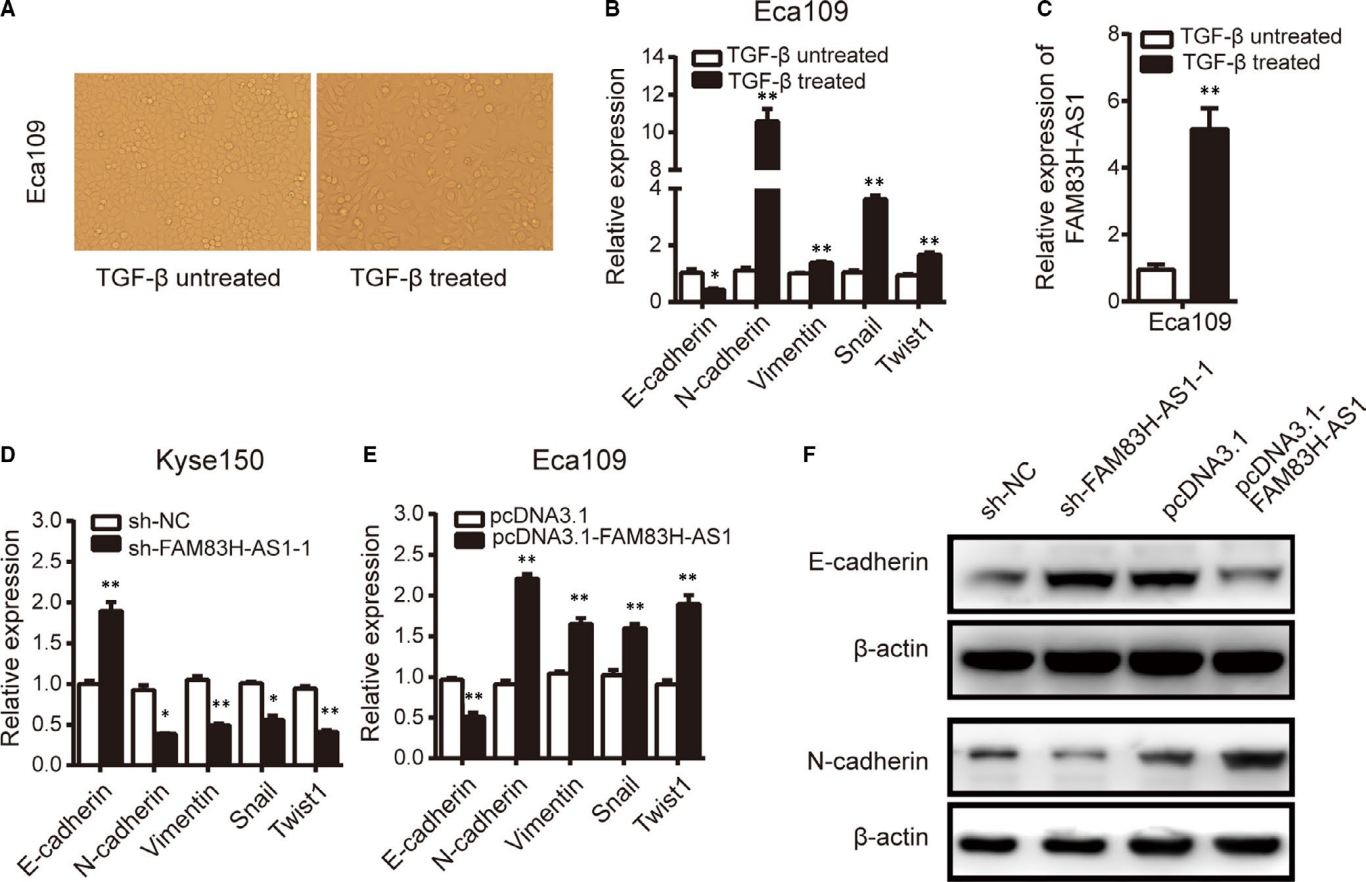
FAM83H‐AS1 is up‐regulated in TGF‐β‐treated Eca109 cells and contributes to EMT process. A, Cell morphology in TGF‐β‐treated or untreated Eca109 cells. B, Relative expression of EMT‐related markers was detected in TGF‐β‐treated Eca109 cells. C, Relative expression of FAM83H‐AS1 was assessed in TGF‐β‐treated Eca109 cells. D, and E, The regulation of FAM83H‐AS1 on EMT‐related markers detected by qRT‐PCR method. F, The effect of FAM83H‐AS1 on EMT‐related markers observed by Western blot assay. Data are shown as mean ± SD; *P < .05 and **P < .01

Because EMT is a crucial step of metastasis, it is of great interest to examine whether FAM83H‐AS1 regulates the migration and invasion of oesophageal cancer cells via EMT. We first measured the cell phenotype after incubation with TGF‐β and found that TGF‐β‐treated Eca109 cells underwent morphological changes to a spindle‐shaped appearance (Figure 4A). Moreover, the cells displayed decreased expression of E‐cadherin, as well as up‐regulated expression of N‐cadherin, vimentin, Snail and Twist1 (Figure 4B). These results suggested that the cells displayed EMT‐associated signatures and exhibited a proper biological response to TGF‐β treatment. Additionally, the expression level of FAM83H‐AS1 was assessed and up‐regulation of FAM83H‐AS1 was detected in TGF‐β‐treated cells compared with untreated cells (Figure 4C). Subsequently, knockdown of FAM83H‐AS1 was found to promote the expression of E‐cadherin and inhibit the expression of N‐cadherin, vimentin, Snail and Twist1 at transcriptional level (Figure 4D), while overexpression of FAM83H‐AS1, compared with negative control, could reduce E‐cadherin expression and enhance expression level of N‐cadherin, vimentin, Snail and Twist1 (Figure 4E). As shown in Figure 4F, FAM83H‐AS1 also regulated EMT‐related markers at protein level. In summary, FAM83H‐AS1 may be an EMT‐related lncRNA and participate in EMT of oesophageal cancer cells.

### 3.6 FAM83H‐AS1 sponges miR‐10a‐5p through direct binding in oesophageal cancer cells

**FIGURE 5:**
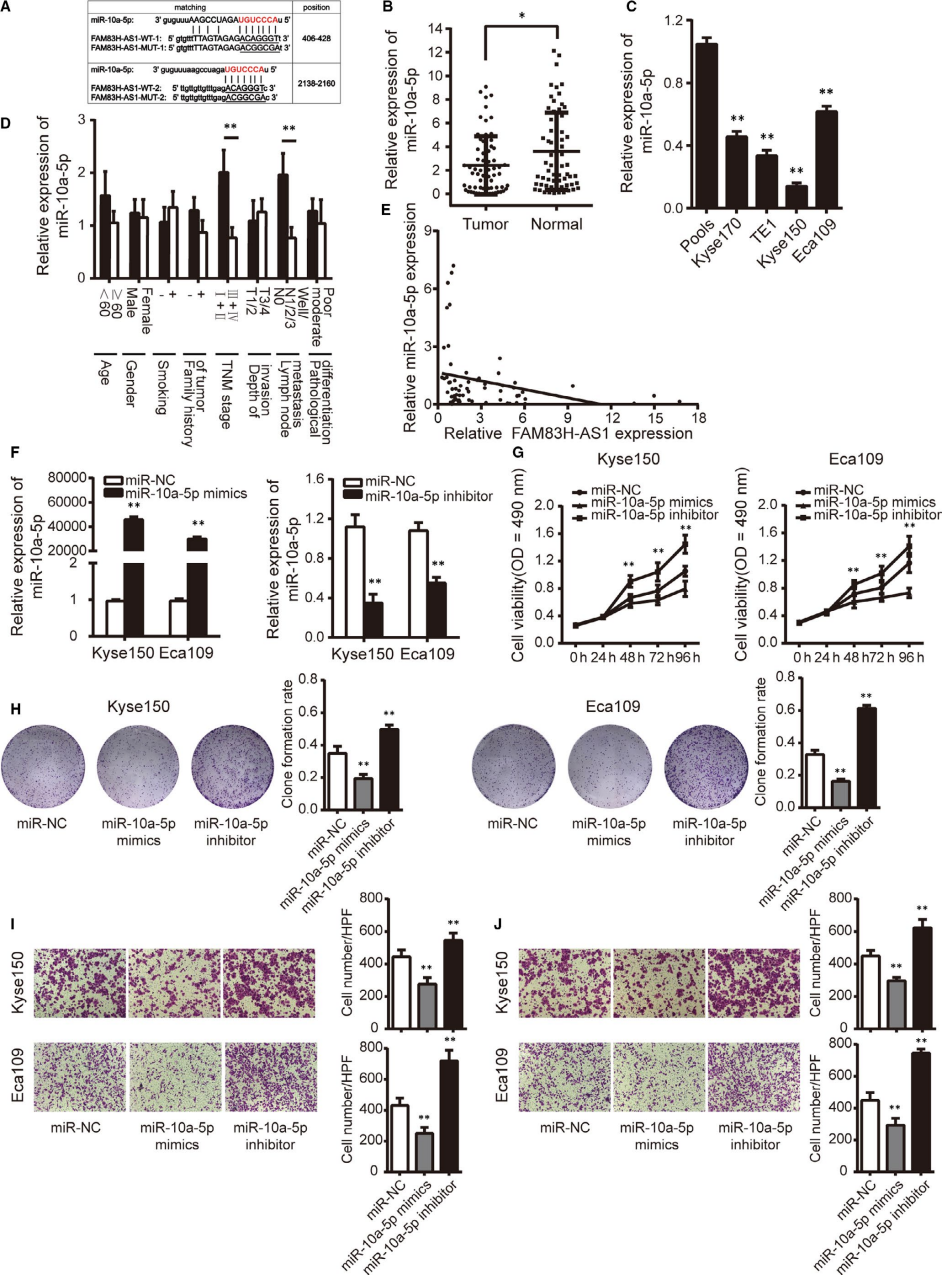
MiR‐10a‐5p contains binding sites to FAM83H‐AS1 and functions as tumour suppressor role. A, The sequence of predicted (wild‐type) and mutated (mutant type) binding sites for miR‐10a‐5p on FAM83H‐AS1. The red nucleotides are the seed sequences of miR‐10a‐5p. B, Relative expression of miR‐10a‐5p in 67 pairs of ESCC tissues and corresponding normal tissues confirmed by qRT‐PCR method. C, Relative expression of miR‐10a‐5p in four human oesophageal cancer cell lines detected by qRT‐PCR method. Pools: average expression in 10 normal tissues was used as normal control. * Compared with the pools. D, Relative expression of miR‐10a‐5p in different subgroups. E, The correlation between FAM83H‐AS1 and miR‐10a‐5p expression. F, Relative expression of miR‐10a‐5p detected by transfection with miR‐10a‐5p mimics or inhibitor. G, MTS assay and H, clone formation assay were conducted by transfection with miR‐10a‐5p mimics or inhibitor. I, Transwell migration and J, invasion assays were performed by transfection with miR‐10a‐5p mimics or inhibitor (magnification, ×200). Data are shown as mean ± SD; *P < .05 and **P < .01

FAM83H‐AS1 was mainly located in the cytoplasm of oesophageal cancer cells; therefore, we hypothesized that FAM83H‐AS1 might also function as a molecular sponge to competitively bind certain miRNAs. According to the prediction in online databases (RAID v2.0), miR‐10a‐5p was found to contain two potential binding sites to the FAM83H‐AS1 sequence (Figure 5A) and chosen for subsequent experiments for its tumour‐suppressive role in ESCC.

The expression level of miR‐10a‐5p was down‐regulated in ESCC tissues and oesophageal cancer cells, as well as closely associated with TNM stage and lymph node metastasis (Figure 5B‐D). In addition, a significant inverse correlation between FAM83H‐AS1 and miR‐10a‐5p expression in ESCC tissues was found (Figure 5E). The efficiency of miR‐10a‐5p mimics and inhibitor was validated prior to further analysis (Figure 5F). As shown in Figure 5G‐J, the overexpression of miR‐10a‐5p by transfection with miR‐10a‐5p mimics hindered the proliferation, migration and invasion of Kyse150 and Eca109 cells, whereas transfection of miR‐10a‐5p inhibitor in Kyse150 and Eca109 cells displayed opposite effects.

**FIGURE 6:**
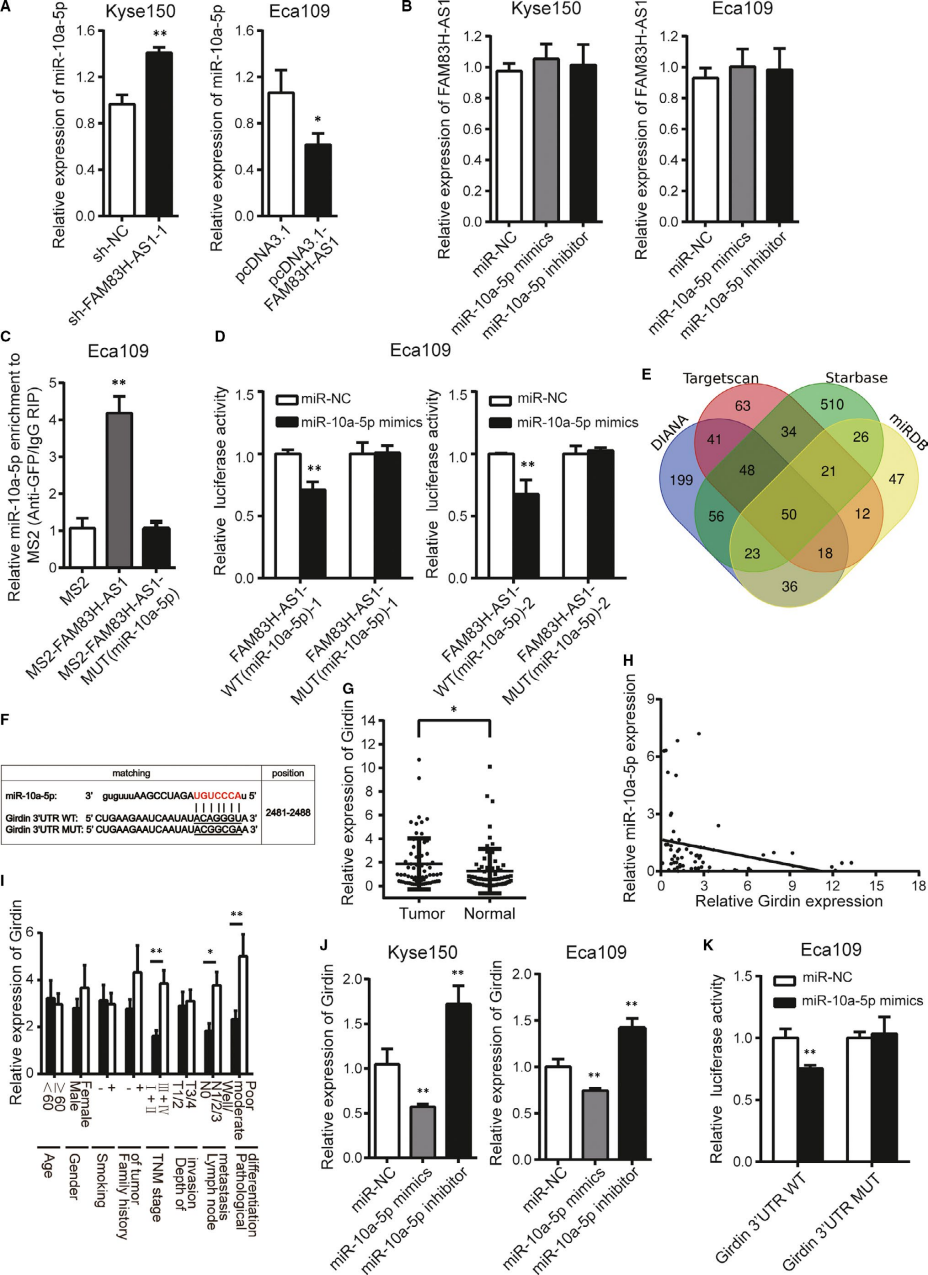
FAM83H‐AS1 sponges miR‐10a‐5p and miR‐10a‐5p directly targets Girdin in oesophageal cancer cells. A, Relative expression of miR‐10a‐5p in FAM83H‐AS1 knockdown or overexpression cells. B, Relative expression of FAM83H‐AS1 in miR‐10a‐5p mimics or inhibitor transfected cells. C, The MS2‐RIP method identified the direct binding between FAM83H‐AS1 and miR‐10a‐5p. D, The effect of miR‐10a‐5p mimics on luciferase activity of wild‐type and mutant‐type FAM83H‐AS1 vectors observed by dual‐luciferase reporter assay. E, The numbers of miR‐10a‐5p targeting the potential same genes (including Girdin) drawn by Venn diagram. F, Schematic representation of the potential binding sites of miR‐10a‐5p on Girdin 3′ UTR. G, Relative expression of Girdin in 67 pairs of ESCC tissues and corresponding normal tissues confirmed by qRT‐PCR method. H, The correlation between Girdin and miR‐10a‐5p expression. I, Relative expression of Girdin in different subgroups. J, The regulation of miR‐10a‐5p on Girdin expression detected by qRT‐PCR method. K, The effect of miR‐10a‐5p mimics on luciferase activity of wild‐type and mutant‐type Girdin 3′ UTR vectors observed by dual‐luciferase reporter assay. Data are shown as mean ± SD; *P < .05 and **P < .01

Furthermore, we explored the relationship between FAM83H‐AS1 and miR‐10a‐5p in Kyse150 and Eca109 cells. FAM83H‐AS1 knockdown resulted in the increase of miR‐10a‐5p expression, and FAM83H‐AS1 overexpression caused visible reductions in miR‐10a‐5p expression (Figure 6A). However, no significant difference in FAM83H‐AS1 expression was found after knockdown or overexpression of miR‐10a‐5p (Figure 6B). The RIP assay was performed to pull down endogenous miRNAs associated with FAM83H‐AS1 and demonstrated that FAM83H‐AS1 significantly enriched with miR‐10a‐5p compared to the empty vector (Figure 6C). Consistently, the relative luciferase activity of FAM83H‐AS1 wild‐type was obviously decreased after cotransfection with miR‐10a‐5p mimics, but did not affect the activity of mutant type, which further verified that miR‐10a‐5p is a direct target of FAM83H‐AS1 (Figure 6D).

### 3.7 miR‐10a‐5p directly targets Girdin in oesophageal cancer cells

By using four independent miRNA target‐predicting algorithms (DIANA, TargetScan, Starbase and miRDB), potential downstream target genes of miR‐10a‐5p were predicted (Figure 6E). Among these 50 predicted target genes, Girdin (also named CCDC88A) attracted our attention because of its critical role in the migration and invasion of cancer cells. Girdin regulates actin reconstruction and Akt‐dependent cell motility, and involves in remodelling actin cytoskeleton which is essential for cell migration.^19^ The conserved binding site of Girdin 3' UTR for miR‐10a‐5p is illustrated in Figure 6F. Girdin expression was found to be higher in ESCC tissues than that in corresponding normal tissues (Figure 6G) and was negatively correlated with miR‐10a‐5p (Figure 6H). Meanwhile, Girdin expression was correlated with TNM stage, lymph node metastasis and pathological differentiation in ESCC tissues (Figure 6I). Subsequently, overexpression of miR‐10a‐5p dramatically decreased the expression level of Girdin, while down‐regulation of miR‐10a‐5p markedly exhibited the opposite effect in oesophageal cancer cells (Figure 6J). Luciferase reporter assay manifested that enforced expression of miR‐10a‐5p reduced the luciferase activity of pmirGLO**‐**Girdin**‐**3′ UTR wild‐type vector while showed no obviously effect on the luciferase activity of pmirGLO**‐**Girdin**‐**3′ UTR mutant type in Eca109 cells (Figure 6K), indicating the indeed regulatory role of miR‐10a‐5p on Girdin mRNA expression through direct binding to its 3′ UTR.

### 3.8 FAM83H‐AS1 positively regulates Girdin in a miR‐10a‐5p‐dependent manner

**FIGURE 7:**
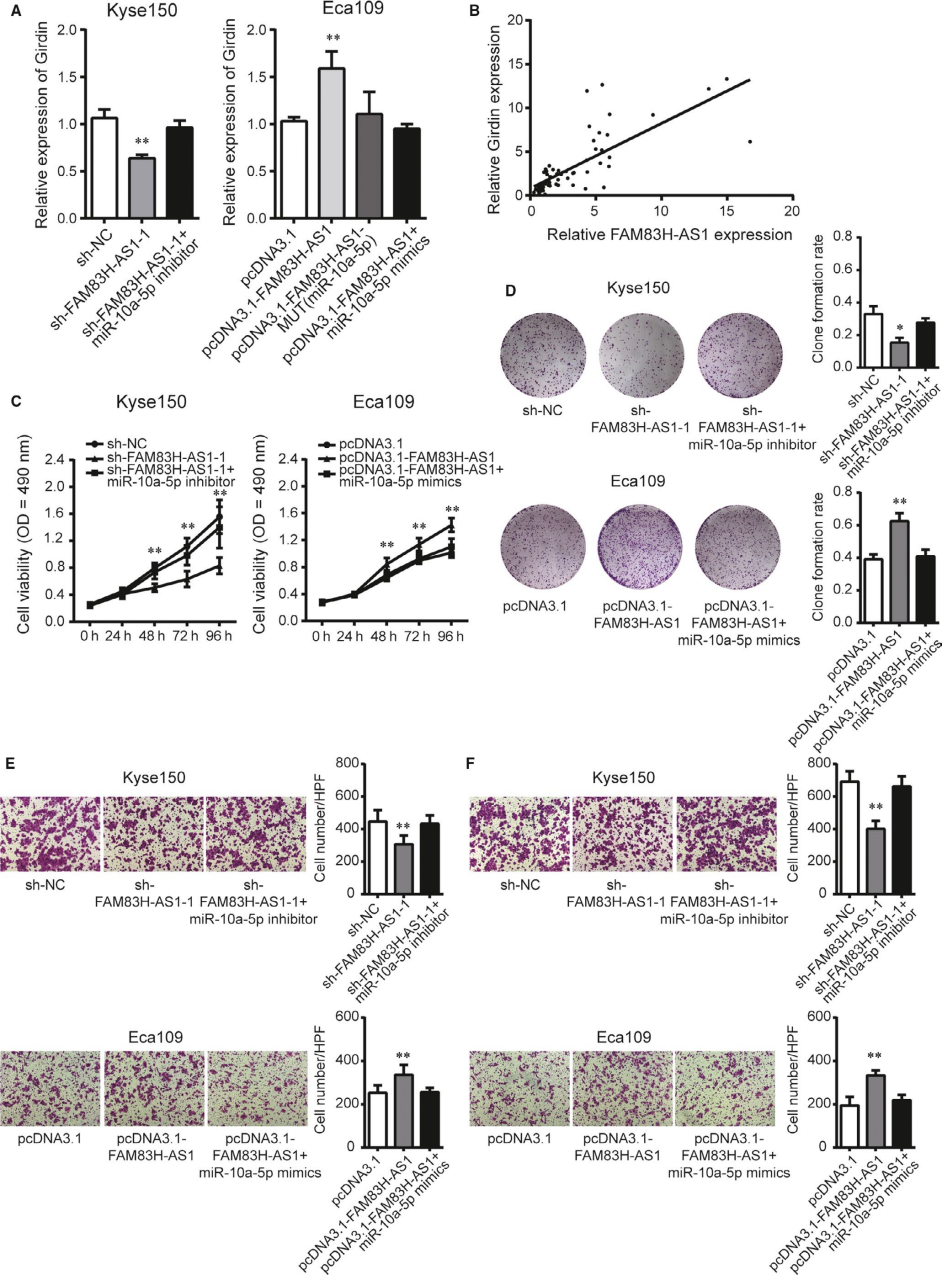
FAM83H‐AS1 positively regulates Girdin in a miR‐10a‐5p‐dependent manner. A, Relative expression of Girdin following cotransfection with miR‐10a‐5p inhibitor or mimics in FAM83H‐AS1 knockdown or overexpression cells. B, The correlation between FAM83H‐AS1 and Girdin expression. C, MTS assay and D, clone formation assay were rescued by cotransfection with miR‐10a‐5p inhibitor or mimics in FAM83H‐AS1 knockdown or overexpression cells. E, Transwell migration and F, invasion assays were confirmed following cotransfection with miR‐10a‐5p inhibitor or mimics in FAM83H‐AS1 knockdown or overexpression cells (magnification, ×200). Data are shown as mean ± SD; *P < .05 and **P < .01

Due to the fact that FAM83H‐AS1 shared common binding sites of miR‐10a‐5p with Girdin, we wondered whether FAM83H‐AS1 could modulate Girdin dependent on miR‐10a‐5p. In oesophageal cancer cells, down‐regulation of FAM83H‐AS1 significantly decreased Girdin expression, whereas miR‐10a‐5p inhibitor overcame such a decrease. Similarly, miR‐10a‐5p mimics could abrogate the increased effect of FAM83H‐AS1 overexpression on Girdin expression (Figure 7A). Besides, a dramatically positive correlation between FAM83H‐AS1 and Girdin expression was identified in 67 pairs of ESCC tissues (Figure 7B). In gain‐ and loss‐of‐function experiments, miR‐10a‐5p inhibitor could partially rescue the inhibitory effect of FAM83H‐AS1 knockdown on cell proliferation, migration and invasion capacity. Reciprocally, miR‐10a‐5p mimics could abolish biological functions caused by FAM83H‐AS1 overexpression (Figure 7C‐F). Overall, these results revealed a vital role of FAM83H‐AS1 in modulating Girdin by competitively binding with miR‐10a‐5p.

## 4 DISCUSSION

There is obvious evidence that the proverbial lncRNAs take up a significant portion operating as either oncogene or tumour suppressor in the pathological development of ESCC. In our study, we verified that FAM83H‐AS1 and its sense transcript FAM83H were consistently up‐regulated, and concordant co‐regulation was detected between these sense‐antisense pairs which possessed as oncogenes in facilitating cell proliferation, migration and invasion. Furthermore, FAM83H‐AS1 promoted the TGF‐β‐induced EMT and functioned as a ceRNA to regulate Girdin expression by competitively binding miR‐10a‐5p in tumorigenesis and progression of ESCC.

NATs are RNA sequences that originate from the opposite DNA strands and partly overlap with sense RNA, promoter or regulatory region. NATs have garnered increased attention on their various regulatory functions ranging from transcriptional regulation to post‐transcriptional regulation. For post‐transcriptional regulation, NATs can regulate gene expression levels through double‐stranded RNA formation, which cause endogenous RNA interference, RNA editing or RNA masking.^20^ In the present study, FAM83H was found to be up‐regulated and positively correlated with FAM83H‐AS1 expression in ESCC tissues. Moreover, FAM83H functioned as tumour promoter by facilitating oesophageal cancer cells viability and invasion, which was consistent with previous studies in cervical cancer and osteosarcomas.^21^, ^22^ Further experiments indicated that FAM83H‐AS1 regulated the expression of FAM83H at transcriptional and post‐transcriptional levels in oesophageal cancer cells. However, it seemed contradictory with the previous results, which was unable to detect changes in FAM83H expression when transfected an siRNA against FAM83H‐AS1.^23^ These phenomena can be interpreted as follows. The functions of lncRNA may present a very stringent cell‐type/tissue specificity, and the different localization of FAM83H‐AS1 resulted in distinct mechanisms in regulating FAM83H. As these data supported a potential oncogenic role of FAM83H in tumorigenesis, further investigation could shed light on the exact mechanisms of concordant expression between FAM83H‐AS1 and FAM83H.

EMT is a fundamental biological process for tumor invasion and metastasis which is characterized by epithelial cells acquiring mesenchymal cells phenotype.^24^ EMT‐initiating transcriptional factors, such as Snail, ZEB1, and Twist1, induce the EMT phenotype that is associated with dramatic expression changes in thousands of genes. Besides, EMT is also characterized by the down‐regulation of epithelial markers (eg E‐cadherin) as well as up‐regulation of mesenchymal markers (eg N‐cadherin, vimentin and fibronectin).^25^, ^26^ Lately, growing evidence has highlighted the TGF*‐*β‐induced lncRNAs implicating in the malignant biological behaviour of cancer by regulating EMT.^27^, ^28^, ^29^ Yuan et al^27^ reported that lncRNA ATB (lncRNA activated by TGF‐β), a mediator of TGF‐β signalling, was crucial for the invasion‐metastasis cascade in hepatocellular carcinoma. Lu et al^28^ observed that TBILA (TGFβ‐induced lncRNA) promoted HGAL expression and bound with S100A7 to enhance its carcinogenic effects in non‐small cell lung cancer. In the present study, FAM83H‐AS1 expression level was higher in TGF‐β‐treated Eca109 cells than control cells, and high level of FAM83H‐AS1 resulted in the up‐regulation of EMT‐initiating transcriptional factors and mesenchymal markers, and the down‐regulation of epithelial markers at mRNA and protein levels, suggesting that FAM83H‐AS1 expression was induced by TGF‐β and promoted EMT in oesophageal cancer metastasis.

LncRNAs involved in versatile aspects of gene regulation and biological processes depending on their subcellular localization. FAM83H‐AS1 was mainly exported to the cytoplasm, where lncRNA could regulate gene expression mainly at post‐transcriptional level. However, Barr et al^23^ reported that FAM83H‐AS1 expression was significant enrichment in the nuclear fractions in comparison with the cytoplasmic fractions, which seemed contradictory with our results. This may be explained by the distinct mechanism of gene in different cancer types. For example, HOXD‐AS1 enriched in the cytoplasm and acted as a ceRNA that sponged up miRNAs to regulate gene expression in ovarian cancer, liver cancer, bladder cancer, non‐small cell lung cancer and glioma in previous studies.^30^, ^31^, ^32^, ^33^, ^34^, ^35^ But in prostate cancer and colorectal cancer, HOXD‐AS1 was determined to be enriched in the nucleus by interacting with critical epigenetic regulators.^36^, ^37^ In the cytoplasm, lncRNAs can regulate mRNA stability via associating miRNAs in a ceRNA manner.^5^ Currently, growing studies revealed that lncRNAs fulfilled their roles as sponging miRNAs to modulate their target mRNA expression and biological functions. For example, Yang et al^38^ found that LINC01133 inhibited gastric cancer progression and metastasis by acting as a ceRNA for miR‐106a‐3p to regulate APC expression and the Wnt/β‐catenin pathway. Huang et al^39^ demonstrated that TRPM2‐AS took important regulatory parts in gastric carcinoma development by functioning as a ceRNA to regulate HMGA1 via sponging miR‐195. However, there are no reports concerning that FAM83H‐AS1 acted as a ceRNA of miRNAs in ESCC, and then we investigated this potential regulatory mechanism of FAM83H‐AS1 in the cytoplasm. In the present study, miR‐10a‐5p was selected as the potential miRNA due to possessing the complementary binding sites with FAM83H‐AS1. MiR‐10a has been previously reported to be a tumour suppressor by analysing the miRNA microarray in ESCC.^40^ Combined with RIP assay and luciferase reporter assay, it confirmed the direct binding between FAM83H‐AS1 and miR‐10a‐5p, implying that FAM83H‐AS1 acted as a molecular sponge of miR‐10a‐5p.

Generally, miRNAs are ubiquitous post‐transcriptional regulators that impact RNA stability and translation rate by binding to mRNAs in a sequence‐specific manner^41^, ^42^, ^43^; therefore, this regulation for target mRNA becomes an important part of the ceRNA network. In this study, Girdin was finally screened out as the specifically target mRNA of miR‐10a‐5p proved by luciferase reporter experiment. Girdin is a novel component of the PI3K/Akt signalling pathway that is a core‐signalling transduction pathway in cancer progression. Previous study has confirmed that Girdin exhibited an enhanced expression in ESCC and presented a positive role in oesophageal cancer cell proliferation, migration and invasion.^44^ Meanwhile, we detected that FAM83H‐AS1 positively regulated Girdin expression abrogated by ectopic expression of miR‐10a‐5p. Taken together, these results supported that FAM83H‐AS1, miR‐10a‐5p and Girdin formed a ceRNA regulatory network in the progression of ESCC.

In conclusion, the current study demonstrated that TGF‐β‐induced FAM83H‐AS1 served as a novel oncogene in ESCC and marked concordant expression with its cognate sense counterpart FAM83H. Additionally, FAM83H‐AS1 was proved to regulate EMT process, and acted as a ceRNA in competitively sponging miR‐10a‐5p to enhance Girdin expression. Furthermore, these findings provided novel insights into the underlying mechanism of the aggressive biological behaviour of ESCC, which highlighted a potential target for ESCC therapy.

## CONFLICT OF INTEREST

The authors confirm that there are no conflicts of interest.

## AUTHOR CONTRIBUTIONS

Wei Guo designed the study and revised the manuscript; Bo Feng performed the experiments, analysed the data and drafted the paper; Gaoyan Wang and Xiaoliang Liang performed the experiments; Zheng Wu and Xinchen Wang prepared the figures and tables; Yanli Guo and Zhiming Dong performed the statistical analysis; and Supeng Shen and Jia Liang recruited the patients and collected the data. All authors read and approved the final manuscript. Bo Feng: Investigation (lead); Software (lead); Writing‐original draft (lead). Gaoyan Wang: Investigation (equal). Xiaoliang Liang: Investigation (equal). Zheng Wu: Software (equal). Xinchen Wang: Software (equal). Zhiming Dong: Software (supporting). Yanli Guo: Software (supporting). Supeng Shen: Methodology (equal). Jia Liang: Methodology (equal). Wei Guo: Writing‐review & editing (lead).

## Supporting information

Supplementary Material

## Data Availability

The data that support the findings of this study are available from the corresponding author upon reasonable request.
