# Supplementary material for: LncRNA FAM83H‐AS1 promotes oesophageal squamous cell carcinoma progression via miR‐10a‐5p/Girdin axis
Source: J Cell Mol Med. 2020 Jun 24;24(16):8962–76. doi: 10.1111/jcmm.15530 (PMC7417701; doi:10.1111/jcmm.15530)
Supplement: Supplementary file 1 — Supplementary Material [file JCMM-24-8962-s001.doc]

**SUPPLEMENTARY FIGURES**


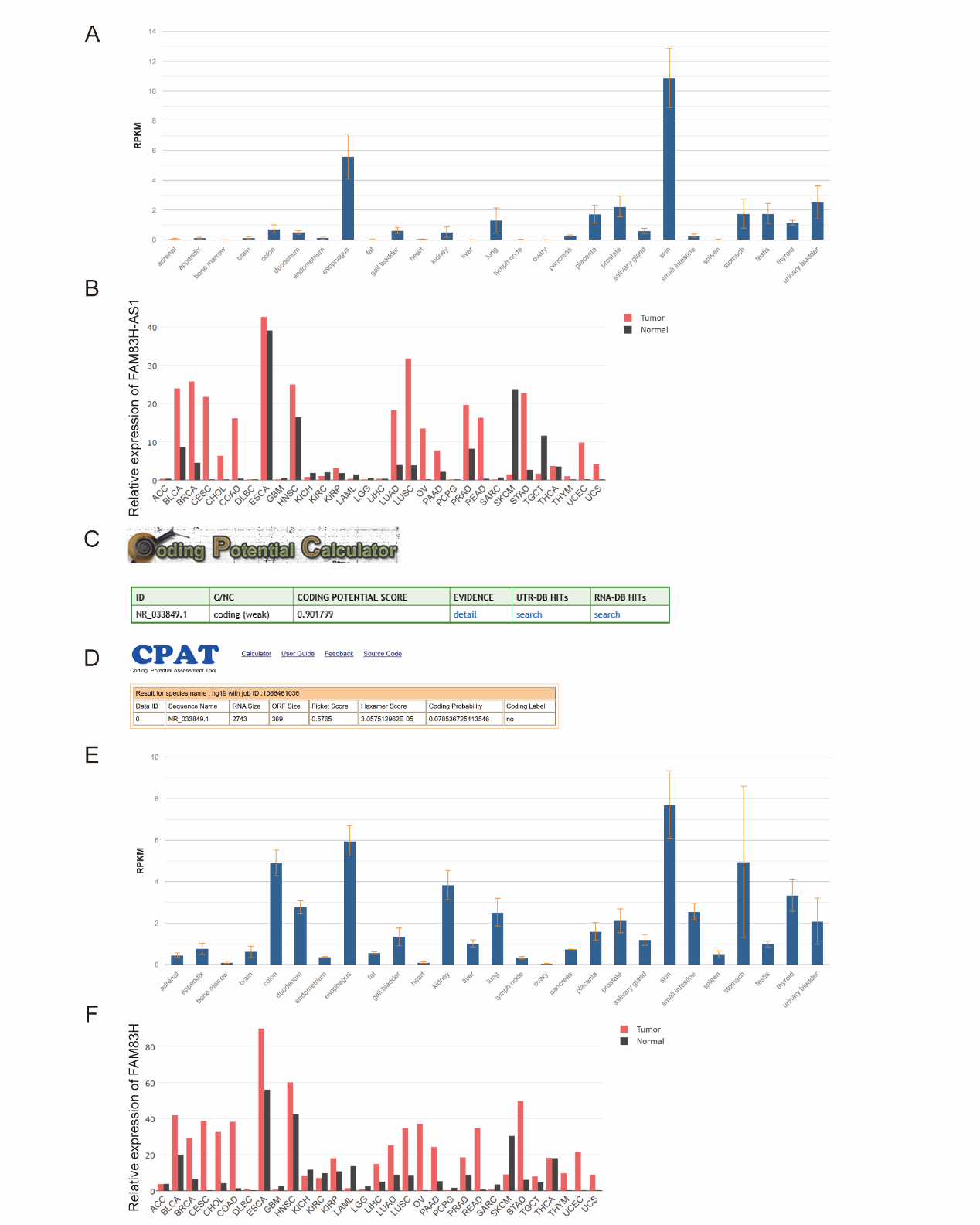


**FIGURE S1** The bioinformatics analysis of FAM83H-AS1 and FAM83H. A, and B, Relative expression of FAM83H-AS1 in different normal tissues referenced from NCBI and in various tumor types cited from GEPIA dataset. C, and D, The coding potential of FAM83H-AS1 predicted by Coding Potential Calculator and Coding Potential Assessment Tool. E, and F, Relative expression of FAM83H in different normal tissues referenced from NCBI and in various tumor types cited from GEPIA dataset.


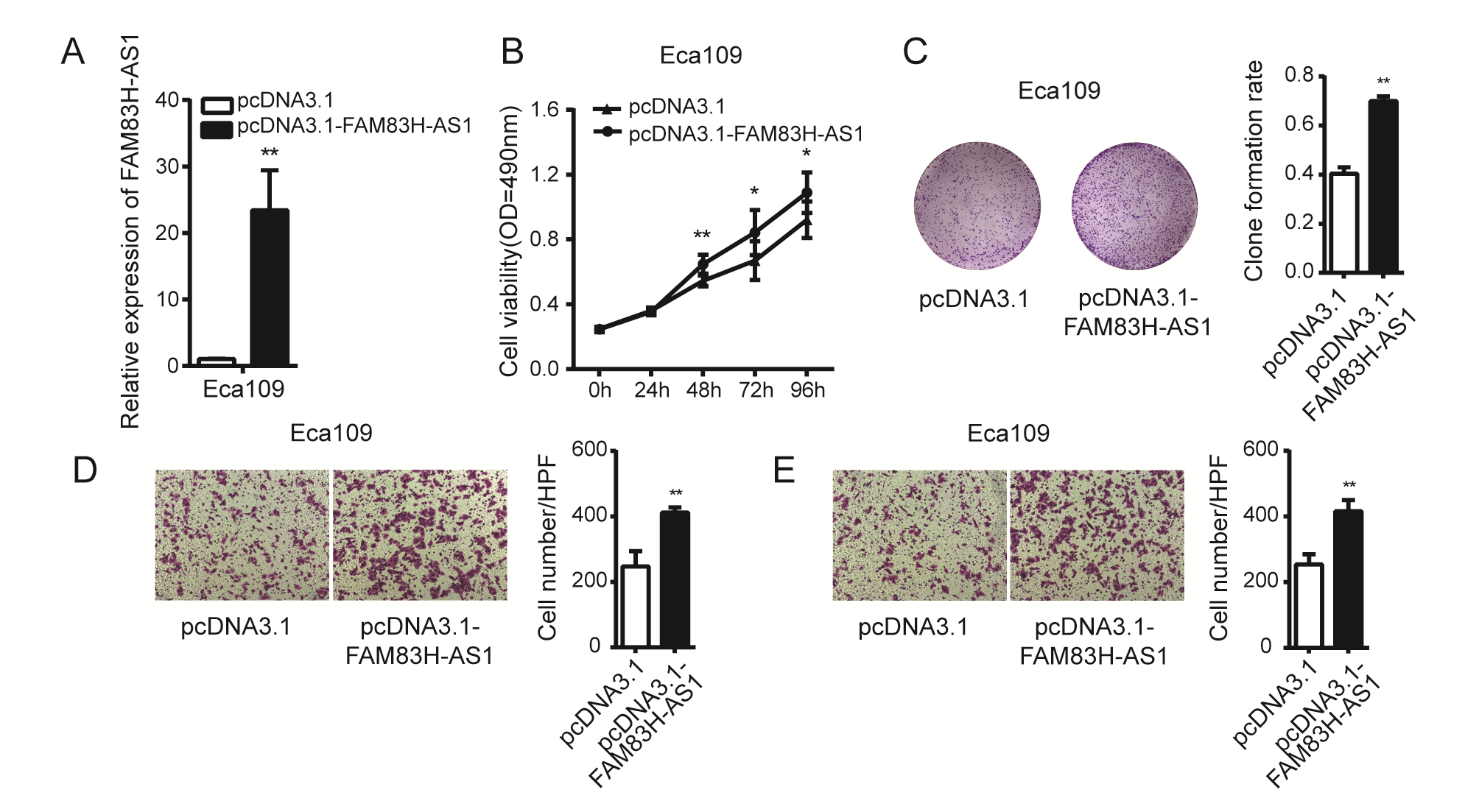
**FIGURE S2** The functional analysis of FAM83H-AS1 overexpression in esophageal cancer cells. A, Overexpression efficiency of FAM83H-AS1 determined by qRT-PCR method. B, MTS assay and C, clone formation assay were conducted with FAM83H-AS1 overexpression in Eca109 cells. D, Transwell migration and E, invasion assays were performed with FAM83H-AS1 overexpression in Eca109 cells (magnification, x200). Data are shown as mean ± SD; * *P* < 0.05 and ** *P* < 0.01.

SUPPLEMENTARY TABLES

**SUPPLEMENTARY TABLE 1** Clinicopathological characteristics of 67 ESCC patients.

| **Characteristics** | | **Number (%)** |
| --- | --- | --- |
| Age (years) |  |  |
|  | <60 | 21(31.3) |
|  | ≥60 | 46(68.7) |
| Gender |  |  |
|  | Male | 48(71.6) |
|  | Female | 19(28.4) |
| Smoking |  |  |
|  | No | 36(53.7) |
|  | Yes | 31(46.3) |
| Family of tumor |  |  |
|  | No | 55(82.1) |
|  | Yes | 12(17.9) |
| TNM stage |  |  |
|  | Ⅰ+Ⅱ | 24(35.8) |
|  | Ⅲ+Ⅳ | 43(64.2) |
| Depth of invasion |  |  |
|  | T1/2 | 18(26.9) |
|  | T3/4 | 49(73.1) |
| Lymph node metastasis |  |  |
|  | N0 | 25(37.3) |
|  | N1/2/3 | 42(62.7) |
| Pathological differentiation |  |  |
|  | Well/moderate | 49(73.1) |
|  | Poor | 18(26.9) |

**SUPPLEMENTARY TABLE 2** Primers used for reverse transcription and qRT-PCR

| **Names** | **Sequences** |
| --- | --- |
| **Reverse Transcription** |  |
| miR-10a-5p stem-loop | 5'-GTCGTATCCAGTGCAGGGTCCGAGGTATTCGCA  CTGGATACGACCACAAATT-3' |
| U6 stem-loop | 5'-AACGCTTCACGAATTTGCGT-3' |
| **qRT-PCR** |  |
| FAM83H-AS1 | F: 5'-CTCACTGGGTCAGCAACACC-3' |
|  | R: 5'-CCCGGGTTGATATTGGGGAAA-3' |
| FAM83H | F: 5'-TTGCAGCACAGTAAAACATG-3' |
|  | R: 5'-GATAAGCACTCCACAACCAG-3' |
| E-cadherin | F: 5'-CGAGAGCTACACGTTCACGG-3' |
|  | R: 5'-GGCCTTTTGACTGTAATCACACC-3' |
| N-cadherin | F: 5'-CAACTTGCCAGAAAACTCCAGG-3' |
|  | R: 5'-ATGAAACCGGGCTATCTGCTC-3' |
| Vimentin | F: 5'-CGCCTGCAGGATGAGATTCAG-3' |
|  | R: 5'-TCAGGGAGGAAAAGTTTGGAAGA-3' |
| Snai1 | F: 5'-ACGAGGTGTGACTAACTAT-3' |
|  | R: 5'-CGACAAGTGACAGCCATT-3' |
| Twist1 | F: 5'-ACCATCCTCACACCTCTG-3' |
|  | R: 5'-GATTGGCACGACCTCTTG-3' |
| miR-10a-5p | F: 5'-GTGCAGGGTCCGAGGTATT-3' |
|  | R: 5'-GCGCTACCCTGTAGATCCG-3' |
| Girdin | F: 5'-AGGAAATGGGACCAACCTTGA-3' |
|  | R: 5'-GTGCATTCTAAGTGAGGCATCAT-3' |
| U6 | F: 5'-CTCGCTTCGGCAGCACA-3' |
|  | R: 5'-AACGCTTCACGAATTTGCGT-3' |
| GAPDH | F: 5'-AGGTGAAGGTCGGAGTCAACG-3' |
|  | R: 5'-AGGGGTCATTGATGGCAACA-3' |

**SUPPLEMENTARY TABLE 3** Primers used for shRNAs, siRNAs, microRNA mimics, and microRNA inhibitor sequences

| **Name** | **Sequences** |
| --- | --- |
| sh-FAM83H-AS1-1 | F: 5'-CACCGGTCTCTGATGTTGGTGTTAATTCAAG  AGATTAACACCAACATCAGAGACCTTTTTTG-3' |
|  | R: 5'-GATCCAAAAAAGGTCTCTGATGTTGGTGTTAA  TCTCTTGAATTAACACCAACATCAGAGACC-3' |
| sh-FAM83H-AS1-2 | F: 5'GACCGATTCAGCTGGCAGCTAAGATTCAAGAG  ATCTTAGCTGCCAGCTGAATGCTTTTTTG-3' |
|  | R: 5'-GATCCAAAAAAGCATTCAGCTGGCAGCTAAGA  TCTCTTGAATCTTAGCTGCCAGCTGAATGC-3' |
| sh-FAM83H-AS1-3 | F: 5'-CACCGCAGCTGTGAGTCTGAATTTCTTCAAGAG  AGAAATTCAGACTCACAGATGCTTTTTTG-3' |
|  | R: 5'-GATCCAAAAAAGCAGCTGTGAGTCTGAATTTCT  CTCTTGAAGAAATTCAGACTCACAGCTGC-3' |
| sh-FAM83H-AS1-4 | F: 5'-CACCGCAACACCCTACTGACCTTGTTTCAAGAG  AACAAGGTCAGTAGGGTGTTGCTTTTTTG-3' |
|  | R: 5'-GATCCAAAAAAGCAACACCCTACAGACCTTGTT  CTCTTGAAACAAGGTCAGTAGGGTGTTGC-3' |
| sh-negative control | F: 5'-CACCGTTCTCCGAACGTGTCACGTCAAGAGATTA  CGTGACACGTTCGGAGAATTTTTTG-3' |
|  | R: 5'-GATCCAAAAAATTCTCCGAACGTGTCACGTAATC  TCTTGACGTGACACGTTCGGAGAAC-3' |
| si-FAM83H-1 | F: 5'-CGGCUUACCCUGAGCGGAATT-3' |
|  | R: 5'-UUCCGCUCAGGGUAAGCCGTT-3' |
| si-FAM83H-2 | F: 5'-UGGCGGAGCUGCUGGAGAATT-3' |
|  | R: 5'-UUCUCCAGCAGCUCCGCCATT-3' |
| si-FAM83H-3 | F: 5'-CGGAGCUGCUGGAGAAGUATT-3' |
|  | R: 5'-UACUUCUCCAGCAGCUCCGTT-3' |
| miR-10a-5p mimics | F: 5'-UACCCUGUAGAUCCGAAUUUGUG-3' |
|  | R: 5'-CAAAUUCGGAUCUACAGGGUAUU -3' |
| negative control | F: 5'-UACCCUGUAGAUCCGAAUUUGUG-3' |
|  | R:5'-CAAAUUCGGAUCUACAGGGUAUU-3' |
| miR-10a-5p inhibitor | 5'-CACAAAUUCGGAUCUACAGGGUA-3'  (2' Ome modulated) |
| inhibitor negative control | 5'-CAGUACUUUUGUGUAGUACAA -3'  (2' Ome modulated) |

**SUPPLEMENTARY TABLE 4** Primers used for vectors construction

| **Names** | **Sequences** |
| --- | --- |
| pmirGLO-FAM83H-AS1-1 (WT) | F: 5'-CGGCTAGCTTGGCTCACTGCTACCTC-3' |
|  | R: 5'-CCCTCGAGGAGGGTCCATGAAAGTGG-3' |
| pmirGLO-FAM83H-AS1-2 (WT) | F: 5'-CTAGCTAGCTGTTGCCCAGGCTGGTCT-3' |
|  | R: 5'-CCGCTCGAGCTGTGTGCCTATAGTCCT-3' |
| pmirGLO-FAM83H-AS1-1 (MUT) | F: 5'-CGATTCACCATGTTGGCCAGG-3' |
|  | R:5'-CCGTCTCTACTAAAAACACAAAAACTAGC-3' |
| pmirGLO-FAM83H-AS1-2 (MUT) | F: 5'-CGACTTTCTCTGTCACCCAGG-3' |
|  | R: 5'-CCGTCTCAAACAACAACAATAAGAAATG-3' |
| pmirGLO-Girdin 3' UTR (WT) | F: 5'-CCCTCGAGAGCCAACTGGAAACGAAT-3' |
|  | R: 5'-GCTCTAGACATGAAAGGTGCTACATAC-3' |
| pmirGLO-Girdin 3' UTR (MUT) | F: 5'-GCTCTAGACATGAAAGGTGCTACATAC-3' |
|  | R: 5'-CCGTATATTGATTCTTCAGTTTCCTTG-3' |
